# Discovering anti-obesity blue food compounds via combined deep learning and in silico approaches

**DOI:** 10.1007/s11030-026-11506-5

**Published:** 2026-03-18

**Authors:** Seo Hyun Shin, Eunseok Oh, Chanyoon Park, Seung Man Oh, Hee Jeong Hwang, Jeong Yun You, Hyeri Ryu, Gihyun Hur, Ji Woo Kim, Jung Han Yoon Park, Eun Roh, Heonjoong Kang, Ki Won Lee

**Affiliations:** 1 Department of Agricultural Biotechnology, Seoul National University, Seoul, 08826 Republic of Korea; 2 Interdisciplinary Graduate Program in Genetic Engineering, Seoul National University, Seoul, 08826 Republic of Korea; 3 Laboratory of Marine Drugs, School of Earth and Environmental Sciences, Seoul National University, Seoul, 08826 Republic of Korea; 4 Bio-MAX Institute, Seoul National University, Seoul, 08826 Republic of Korea; 5 Research Institute of Oceanography, Seoul National University, Seoul, 08826 Republic of Korea; 6 Research Institute of Agriculture and Life Sciences, Seoul National University, Seoul, 08826 Republic of Korea

**Keywords:** Blue food, Deep learning, Food compounds, AMP-activated protein kinase (AMPK), Anti-obesity

## Abstract

Blue foods, such as fish and aquatic sources, are rich in bioactive compounds with potential health benefits, including anti-obesity effects. AMPK, a key regulator of energy homeostasis and adipogenesis, plays a vital role in combating obesity by inhibiting adipocyte differentiation. This study aimed to identify natural AMPK activators from underexplored blue foods using a deep learning-based compound-protein interaction (CPI) prediction model that incorporates both local and global features of proteins and compounds. Local and global molecular characteristics were systematically represented using sequence-based encodings, molecular fingerprints, and protein descriptors to enhance the model’s ability to capture diverse biochemical relationships. The deep learning framework integrated these multimodal features through neural network modules to predict potential compound–protein interactions. In silico molecular docking was performed to refine the predicted interactions, leading to the identification of 94 top-scoring compounds with strong binding affinity toward AMPK. In vitro validation of five commercially available compounds confirmed anti-obesity activity. Notably, cyclo(Pro-Val) from *Ulva pertusa*, activated AMPK and modulated key adipogenic markers, suggesting its potential as a natural AMPK activator for obesity prevention and the feasibility of AI-driven screening for marine bioactives.

**Supplementary Information:**

The online version contains supplementary material available at 10.1007/s11030-026-11506-5.

## Introduction

Blue foods, defined by Gephart et al. [1] as fish and other aquatic foods, have captured significant attention recently. They offer a range of benefits: they are rich in essential nutrients, have a lower environmental impact compared to terrestrial agriculture, and support sustainable food systems [1, 2]. Natural compounds derived from these marine sources have shown promising biological effects, such as anti-diabetic, anti-inflammatory, antioxidant, and anticancer properties [3]. Despite their potential, research on these compounds is less comprehensive compared to terrestrial plants. This quest for bioactive compounds from blue foods opens up exciting possibilities for the future of health and nutrition.

Obesity is a chronic disease characterized by excessive adiposity that can impair health [4]. Obesity poses a significant threat to global health, with projection estimating over one billion obese adults by 2030 [5]. The increasing prevalence of obesity amplifies the risk of noncommunicable diseases (NCDs), including diabetes, heart disease, cancer, and chronic respiratory issues [4]. Current anti-obesity medications approved by Food and Drug Administration (FDA) of the United States are Orlistat, Phentermine/Topiramate, Naltrexone/bupropion, Liraglutide, and Semaglutide [6]. However, these medications come with the risk of various side effects, including headaches, abdominal pain, vomiting, nausea, dizziness, diarrhea, cardiovascular abnormalities, insomnia, depression, and more [7]. Additionally, glucagon-like peptide 1 agonists, typically prescribed for diabetes management, are being used off-label for obesity treatment. However, patients with diabetes may experience gastrointestinal adverse events, including biliary disease, pancreatitis, bowel obstruction, and gastroparesis [8]. Due to the side effects of anti-obesity medications, research is needed to discover safer alternatives derived from natural products.

As reviewed by Steinberg et al. [9], AMP-activated protein kinase (AMPK) stands out as a crucial target protein linked to obesity, and its relevance is underscored by its association with metformin, a widely prescribed antidiabetic medication [10]. The metabolic process of living cells utilizes ATP and ADP as energy sources, generating AMP in the process. AMPK, a serine/threonine kinase, plays a pivotal role as a fuel-sensing enzyme. AMPK is activated by an increase in AMP during cellular energy expenditure, inhibiting ATP usage and inducing catabolic processes to maintain energy homeostasis [9].

Serving as a fuel sensor, AMPK plays a role in controlling energy homeostasis. Activation of AMPK has been shown to exert inhibitory effects on adipogenesis, influencing the differentiation and maturation of adipocytes. AMPK activates SIRT1 and modulates the Wnt/β-catenin pathway by increasing the expression and nuclear accumulation of β-catenin. Consequently, this activation leads to the inhibition of gene expressions associated with adipogenesis, including CCAAT/enhancer-binding protein alpha (C/EBPβ), Peroxisome proliferator-activated receptor (PPARγ), CCAAT/enhancer-binding protein alpha (C/EBPα), Fas Cell Surface Death Receptor (FAS), adipocyte Protein 2 (aP2), and Sterol regulatory element binding protein 1c (SREBP-1c) [11, 12]. The suppression of expression of these key genes results in the inhibition of adipogenesis. Furthermore, AMPK induces G1 cell-cycle arrest by down-regulating Cyclin D1 and retinoblastoma protein (pRb), consequently inhibiting early adipogenesis in the mitotic clonal expansion (MCE) phase [11, 13].

AMPK is heterotrimeric and comprises three subunits: a catalytic subunit α, and regulatory subunits β and γ in various isoforms (α1, α2, β1, β2, γ1, γ2, γ3). As reported by Wang et al. [12], the knockout of both AMPK α1 and α2 subunits in adipose tissue (AMPK α1 α2-AKO) leads to a reduction in basal lipolysis and an increase in lipolysis when adipose tissue is exposed to isoproterenol. This underscores the essential role of AMPKα in adipose tissue thermogenesis and energy expenditure. Additionally, Villena et al. [14] observed elevated body weight and fat mass in mice lacking the AMPK α2 subunit. In the study conducted by Wang et al. [15], it was highlighted that adipogenesis was more significantly suppressed in 3T3-L1 cells overexpressing α2 compared to α1. Hence, our research aims to identify a Blue foods-derived AMPK activator specifically tailored to target the α2 subunit.

The traditional search for natural products that specifically target proteins has predominantly relied on experimental methods [16]. However, these conventional approaches are often associated with significant challenges, including high costs, lengthy timelines, and relatively low success rates. For instance, the discovery and development of a single drug typically require an estimated US$2.8 billion and approximately 15 years of research and development [17]. Recent advancements in computational methods, however, have revolutionized the field of natural product discovery. These innovations have dramatically reduced both the financial and temporal burdens of drug discovery [18]. Cutting-edge techniques, such as machine learning-based drug discovery, in silico molecular docking, and molecular descriptor analyses, have significantly streamlined the identification and characterization of bioactive natural compounds [19, 20].

Recently, CNN models, a type of deep learning framework, have been actively utilized in the discovery of active compounds. These CNN models extract representative features from training data, known as convolutional features, and have been shown to outperform traditional machine learning models. However, they primarily focus on local patterns, lacking global information [21, 22]. This limitation is particularly significant in drug-target interactions, where both global and local features contribute to interaction scores. For both compounds and proteins, structural elements that are distant in atom or amino acid sequence could be spatially close in binding space and play critical roles in compound-protein interactions (CPI). To address these limitations, we developed a model that incorporates molecular descriptors and protein descriptors, which are global features derived from the overall structures of compounds and proteins. By integrating global information with local patterns, our approach is expected to enhance the predictive performance of CNN-based models in identifying CPIs.

In this study, we developed an integrated approach combining deep learning and in silico methods to efficiently identify novel bioactive compounds with potential anti-obesity effects from the natural product repository of blue foods. This approach was focused on discovering activators of the AMPK, a key protein implicated in the regulation of obesity and diabetes. To achieve this goal, we trained a deep learning model to predict CPI using local and global features of compounds and proteins, thereby maximizing the integration of available structural and functional information. The trained model was then employed to predict CPI values for blue food compounds targeting the AMPK a2 subunit. Based on these predictions, in silico molecular docking was performed for the top 10% of compounds with the highest predicted CPI values, resulting in the identification of 94 promising candidates. Among these, five commercially available compounds were selected for in vitro validation, all of which demonstrated significant inhibition of adipogenesis.

## Materials and methods

### Deep learning model construction

#### Overview of the model

**Fig. 1:**
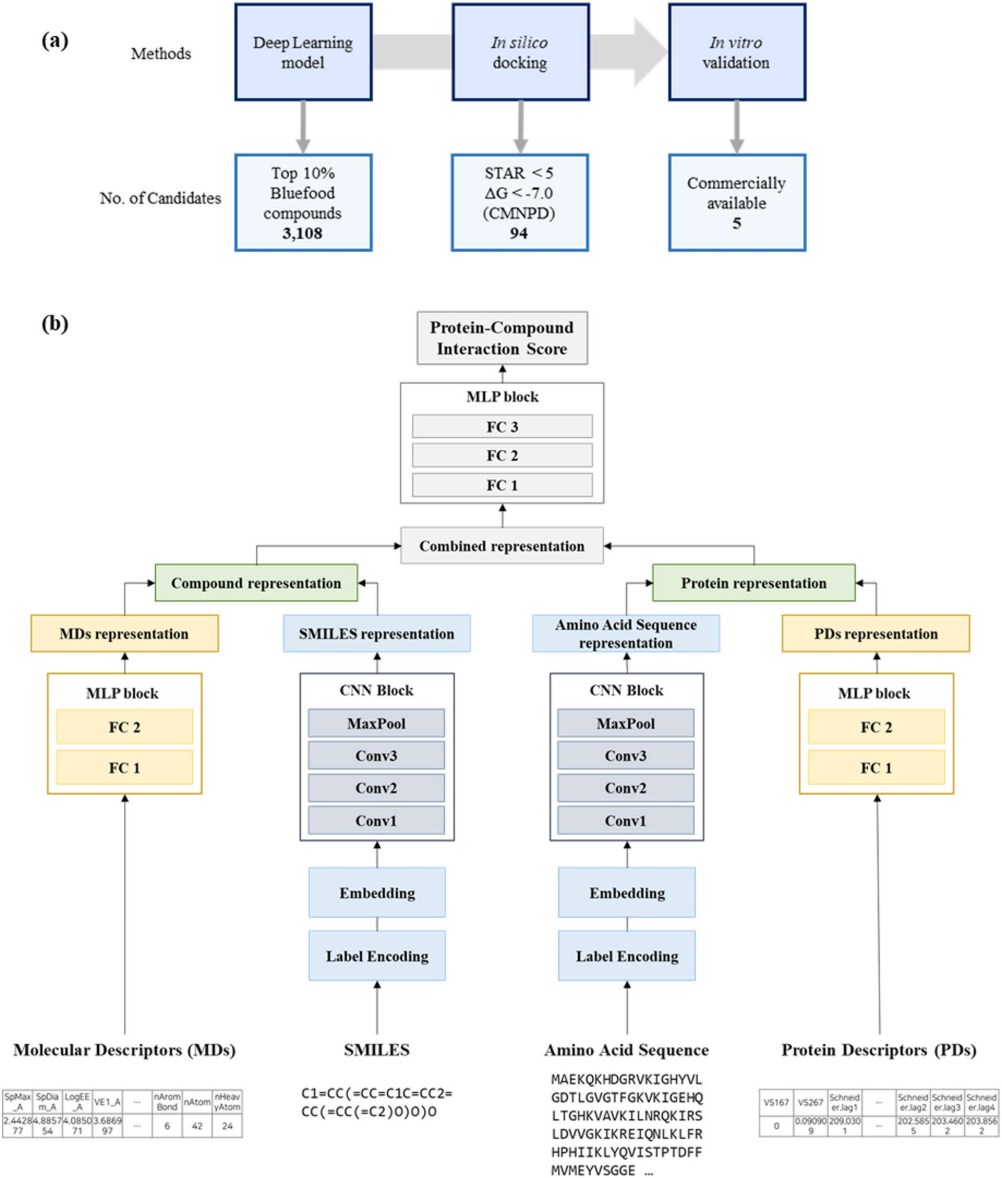
**a** The workflow of the combined deep learning approach and in silico approach to discover anti-obesity blue food compounds and **b** the architecture of the deep learning approach. The deep learning model utilizes local and global information of both compounds and proteins to predict CPI values

In this study, we aimed to build a machine learning model to predict CPI using local and global features of both compounds and proteins. The overall schematic of proposed model is depicted in Fig. 1. To extract local features for compounds and proteins, a CNN architecture was employed. This choice stems from the suitability of CNNs in capturing local patterns within data, making them particularly well-suited for extracting local features. In this context, CNNs excel at recognizing spatial hierarchies and patterns within the compound and protein structures, contributing to effective local feature extraction [23]. For obtaining global features, molecular descriptors suitable for representing compound and protein global features were computed individually. These descriptor values were then utilized as inputs for fully-connected layers, allowing the generation of a comprehensive representation for global features. Subsequently, the local and global features of compounds and proteins were concatenated into a combined representation. This combined representation was leveraged through additional fully-connected layers to predict the CPI value.

Utilizing transfer learning techniques often leads to improved performance in various machine learning tasks. This approach involves initially training the model on a more extensive dataset before fine-tuning it on the specific target dataset [24]. In this study, we adopted a two-step training strategy: the model was first pre-trained on the KIBA dataset [25], a benchmark CPI dataset, and subsequently fine-tuned via transfer learning on the AMPK dataset, which is comparatively smaller in size.

#### Data preparation

To pre-train a model predicting CPI using both local and global features of compounds and proteins, the Kiba dataset was employed as a benchmark CPI dataset. The Kiba dataset consolidates kinase inhibitor activity data gathered from various sources, providing Kiba scores defined using Ki, Kd, and IC_50_ values. In this study, the Kiba dataset curated by He et al. [26] was utilized, which encompasses 118,254 CPI entries between 229 proteins and 2,111 drugs.

For transfer learning, an associated CPI dataset specific to predicting interactions with the AMPK a2 subunit was collected from ChEMBL [27]. A total of 245 EC_50_ data points for AMPK a2 subunit were gathered, and the EC_50_ values were transformed into log space, ultimately using pEC_50_ values in the model.

Blue foods-derived natural products data was obtained from the Comprehensive Marine Natural Products Database (CMNPD) [28]. CMNPD is a significant resource for the research and discovery of bioactive compounds from blue foods. The database currently contains detailed information on over 32,000 marine source derived-compounds, including their trivial and systematic names, chemical structure, and physicochemical and pharmacokinetic properties. After removal of duplicates, we obtained 31,080 chemical entities along with their respective compound information.

#### Compound representation

To extract local features for compounds, the SMILES string of each compound was utilized. SMILES is a notation that represents the structure of a chemical compound in a concise and human-readable string format. In this study, the SMILES of compounds were subjected to label encoding, transforming them into integer representations. These encoded SMILES were adjusted to a fixed length of 100 characters. SMILES strings exceeding 100 characters were truncated, while those shorter than 100 characters were zero-padded to achieve uniform length.

For the extraction of global features for compounds, molecular fingerprinting and molecular descriptors were employed. The molecular fingerprint was computed using the Extended-Connectivity Fingerprints (ECFP4) with 1,024 dimensions from RCDK [29]. Additionally, molecular descriptors were computed using Mordred [30], resulting in a set of 1540 descriptors, after excluding descriptors with null values.

#### Protein representation

To extract local features of the protein, its amino acid sequence of the protein was utilized. The amino acid sequence was converted into integer format through label encoding, following the methodology outlined in prior research [21]. The sequence length was standardized to 1,000 by truncating sequences longer than 1000 and applying zero-padding to sequences shorter than 1,000.

For the extraction of global features, protein descriptors were calculated using the Protr package [31]. Protr generates 9,920 protein descriptors, capturing diverse properties such as amino acid composition, autocorrelation, CTD (Composition, Transition, Distribution), quasi-sequence-order, and pseudo-amino acid composition.

#### Model construction and evaluation

This study was inspired by deepDTA [21] and developed a modified deepDTA model to predict interactions between chemicals and proteins. The model features four specialized heads, each designed to process specific information: local and global features of compounds and proteins. Each head employs appropriate neural network architectures to extract these features effectively. The outputs from the four heads are concatenated (total 1,216 dimension) and passed through a final fully connected layer. The model is trained to minimize the difference between predicted and actual CPI values, Kiba score or pEC_50_. The implementation was carried out using the PyTorch framework.

The model was trained and evaluated on a comprehensive dataset of known chemical-protein interactions with corresponding CPI values. The dataset was divided into six subsets: one-sixth served as the test set, while the remaining data were used for 5-fold cross-validation. The mean squared error (MSE) loss function was employed to quantify the difference between predicted and true CPI values. Model parameters were optimized using the Adam optimizer.

Model performance was assessed on the test set using metrics such as Mean Squared Error (MSE), Concordance Index (CI), R-squared (R^2^), and Pearson Correlation Coefficient (PCC). The metrics are based on the difference between the observed CPI $y$ (Kiba score or pEC_50_) and the predicted CPI $\widehat{y}$, where m is the member of protiens. MSE (1) is a measure of the average squared difference between the predicted and actual values, and lower MSE indicates better accuracy.

1 $$\mathrm{M}\mathrm{S}\mathrm{E}\left(y,\widehat{y}\right)=\frac{{\sum}_{i}{\left({y}_{i}-\widehat{{y}_{i}}\right)}^{2}}{m}$$

CI (2) is a measure of the pairwise ranking consistency between predicted and observed values, and can be interpreted as the probability that, in a randomly selected pair of samples, the one with the higher predicted value has the higher actual value. The metric is described in the following form, where $\widehat{{y}_{i}}$ is the predicted CPI value for the larger CPI value ${y}_{i}$, $\widehat{{y}_{j}}$is the predicted CPI for the smaller CPI value ${y}_{j}$, Z is a normalization constant, and $h\left(x\right)$ is the step function:

2 $$\mathrm{C}\mathrm{I}=\frac{{\sum}_{{y}_{i}>{y}_{j}}h\left(\widehat{{y}_{i}}-\widehat{{y}_{j}}\right)}{Z},h\left(x\right)=\left\{\begin{array}{c}1,ifx>0\\0.5,ifx=0\\0,ifx<0\end{array}\right.$$

R-squared (3), or the coefficient of determination, indicates the proportion of variance in the dependent variable that is predictable from the independent variables, with higher values indicating that more variance is explained by the model.

3 $${R}^{2}=1-\frac{{\sum}_{i}{\left({y}_{i}-\widehat{{y}_{i}}\right)}^{2}}{{\sum}_{i}{\left({y}_{i}-\stackrel{-}{{y}_{i}}\right)}^{2}}$$

PCC measures the linear correlation between the two variables. PCC can be described as (4), while $\mu\left(x\right)$is the mean of a random variable. Both provide measures of correlation that measures the strength and direction of the relationship between actual and predicted value of the model.

4 $$\mathrm{P}\mathrm{C}\mathrm{C}\left(y,\widehat{y}\right)=\frac{{\sum}_{i}\left(\widehat{{y}_{i}}-\mu\left(\widehat{y}\right)\right)\left({y}_{i}-\mu\left(y\right)\right)}{\sqrt{{\sum}_{i}{\left(\widehat{{y}_{i}}-\mu\left(\widehat{y}\right)\right)}^{2}}\sqrt{{\sum}_{i}{\left({y}_{i}-\mu\left(y\right)\right)}^{2}}}$$

### In silico approach

The chemical structures of the selected compounds were prepared using Schrödinger’s LigPrep software [32]. The OPLS4 force field was applied to generate tautomers and possible states at a pH range of 7.0 ± 2.0. The three-dimensional structure of the AMPK protein was obtained from the Protein Data Bank (PDB:4ZHX) [33]. Protein preprocessing steps included the removal of water molecules, addition of hydrogen atoms, and refinement through hydrogen bond assignment and restrained energy minimization. To generate the protein grid, a box was centered around the selected binding site residues: Lys29, Lys31, Arg83, Asp88, and Ser108 [34, 35]. The grid box was set to accommodate ligands within a 20 Å radius. Ligand docking was performed utilizing the Glide module from Schrödinger, employing high-throughput virtual screening (HTVS) precision [36]. Additionally, the molecular properties such as absorption, distribution, metabolism, and excretion (ADME) of the ligands were assessed using QikProp software [37] and SwissADME [38].

### Validation of predicted blue food compounds

#### Chemicals and materials

The following chemicals and materials were sourced from the indicated suppliers: methyl 4-bromopyrrole-2-carboxylate, cyclo(Pro-Leu), and isatin (BLDpharm, Shanghai, China); cyclo(Pro-Val) (Cayman Chemical, Ann Arbor, MI, USA). All blue food compounds were dissolved in dimethyl sulfoxide (DMSO; Sigma Aldrich).

#### Validation of anti-adipogenic effects: Adipocyte differentiation of 3T3-L1 preadipocytes

3T3-L1 preadipocytes, purchased from ATCC (Manassas, VA, USA), were cultured at 37 °C in a controlled environment with 5% CO_2_, using Dulbecco’s Modified Eagle’s Medium (DMEM; Welgene Inc., Daegu, South Korea) supplemented with 10% bovine calf serum (Sigma Aldrich), 100 units/mL of penicillin, and 100 µg/mL of streptomycin (Gibco, Grand Island, NY, USA). Upon reaching full confluence (Day 0), the preadipocytes were induced to differentiate using differentiation medium (DM) consisting of DMEM supplemented with 10% fetal bovine serum (FBS; Gibco), 10 µg/mL human insulin (Sigma-Aldrich), 0.5 mM isobutylmethylxanthine (Sigma-Aldrich), and 1 µM dexamethasone (Sigma-Aldrich) for two days. Following this initial induction, cells were cultured in post-differentiation medium (post-DM), which contained DMEM, 10% FBS, and 10 µg/mL insulin for additional two days. The medium was then replaced with maintenance medium containing DMEM and 10% FBS for three more days to complete the differentiation process. To evaluate the anti-adipogenic effects of selected blue food compounds, these compounds were added to the culture medium simultaneously with the differentiation medium on Day 0 and maintained throughout the entire differentiation process until Oil Red O staining was performed on Day 7. Lipid accumulation, assessed via Oil Red O staining, was quantified as a measure of adipocyte differentiation inhibition.

#### Oil red O staining

Following adipocyte differentiation, Oil Red O staining was performed to visualize and quantify intracellular lipid accumulation, a key marker of adipocyte maturation. On Day 7, cells were fixed with 10% formalin for 1 h and subsequently washed with 60% isopropanol. The fixed cells were then stained with Oil Red O solution for 15 min at room temperature to selectively label intracellular lipid droplets. Excess stain was removed by washing with distilled water, and the stained cells were observed under an optical microscope. The presence and intensity of red staining served as both qualitative and quantitative indicators of adipocyte differentiation and lipid accumulation.

For quantitative analysis, retained Oil Red O was extracted using 100% isopropanol, and the absorbance of the eluted solution was measured at 515 nm using a spectrophotometer. This absorbance value provided a quantitative assessment of lipid accumulation, which was analyzed to evaluate the efficacy of blue food compounds in inhibiting adipogenesis in 3T3-L1 cells. Additionally, to confirm that the observed effects were specific for inhibition of adipogenesis rather than cytotoxicity, post-staining cell viability analysis was performed using the MTT assay.

#### Quantitative real-time (qRT) polymerase chain reaction (PCR)

Total RNA was isolated from cells on Day 7 using TRIzol reagent (Invitrogen) following the manufacturer’s protocol. Complementary DNA (cDNA) was synthesized using a reverse transcriptase kit (K1622, Thermo Scientific) according to the manufacturer’s instructions. Gene-specific primers were synthesized by Cosmogenetech (Seoul, Korea) and are listed in Table 1. Quantitative real-time PCR (qRT-PCR) was performed using a CFX96 real-time PCR detection system (Bio-Rad Laboratories, USA). The relative mRNA expression levels were calculated using the 2 − ΔΔCt method [39], with β-actin as the internal control.

**Table 1:** Primer sequences used for quantitative real-time PCR gene expression analysis

Gene	Forward primer sequences (5’->3’)	Reverse primer sequences (5’->3’)
*ADIPOQ*	GGCAGGAAAGGAGAACCTGG	AGCCTTGTCCTTCTTGAAGAG
*β-actin*	TTCTTTGCAGCTCCTTCGTT	ATGGAGGGGAATACAGCCC
*CD36*	CAGTCGGAGACATGCTTATTGAG	TTTGCCACGTCATCTGGGTTT
*CEBPA*	CAAGAACAGCAACGAGTACCG	GTCACTGGTCAACTCCAGCAC
*FABP4*	AAGAAGTGGGAGTGGGCTTT	GCTCTTCACCTTCCTGTCGT
*GLUT4*	TCATTGTCGGCATGGGTTT	CGGCAAATAGAAGGAAGACGTA
*PPARG2*	GCATGGTGCCTTCGCTGA	TGGCATCTCTGTGTCAACCATG

#### Western blot assay

Western blot analysis was performed according to our previously established protocol [40]. Briefly, 3T3-L1 preadipocytes were cultured in DMEM supplemented with 10% BCS for two days. Upon reaching confluence, the medium was replaced with DM, with or without cyclo(Pro-Val). On Day 7, cell lysates were collected, and protein concentrations were determined using a dye-binding protein assay kit (Bio-Rad Laboratories, Hercules, CA, USA) following the manufacturer’s instructions. The lysates were then subjected to sodium dodecyl sulfate-polyacrylamide gel electrophoresis (SDS-PAGE) on 10% gels and subsequently transferred onto polyvinylidene difluoride (PVDF) membranes (GE Healthcare, Chicago, IL, USA). Following blocking with 5% skim milk, the membranes were incubated with specific primary antibodies(phospho-AMPKα2(Thr172), AMPKα2, PPARγ and C/EBPα; Cell Signaling Technology, MA, USA)), followed by horseradish peroxidase-conjugated secondary antibodies(GenDEPOT, TX, USA). Protein bands were visualized using a chemiluminescence detection kit (Amersham Pharmacia Biotech, Little Chalfont, UK).

#### AMPK activity assay

AMPK activity was measured using the AMPK (α2β1γ1) kinase enzyme system in combination with the ADP-Glo™ assay kit (Promega) in 96-well plates with a final reaction volume of 40 µL, following the manufacturer’s protocol. Each reaction mixture contained 25 ng of AMPK enzyme, 5 µg of SAMS peptide, and 2.5 µM AMP, prepared in the presence or absence of the test ligand. The reaction was initiated by adding 250 µM ATP and allowed to proceed for 15 min at 30 °C. Enzymatic activity was terminated by the addition of ADP-Glo™ reagent, followed by a 40-min incubation at room temperature. Subsequently, kinase detection reagent was introduced and incubated for an additional 30 min. Luminescence signals were then measured using a Victor Nivo multimode microplate reader (PerkinElmer, Waltham, MA, USA).

#### Statistical analysis

Results are presented as mean ± standard error of the mean (SEM). Statistical significance was assessed using an unpaired two-tailed Student’s t-test or one- or two-way ANOVA, followed by Tukey’s post hoc test where applicable. A P values of less than 0.05 was considered statistically significant. All statistical analyses were performed using GraphPad Prism version 10 (GraphPad Software, USA).

## Results

### Performance metrics demonstrating the predictive power of the proposed deep learning model

The pre-trained model, trained on the Kiba dataset to predict CPI values, achieved an MSE of 0.209, a CI of 0.844, an R² value of 0.693, and a PCC of 0.837. These metrics collectively indicate that the model performed well in capturing the relationships between compounds and proteins within the Kiba dataset. The primary objective of this study is to identify potential candidate compounds for subsequent validation. Therefore, the CI, which measures the relative predictive performance, is considered the most critical performance metric. When the model was transfer-learned to the AMPK dataset, the MSE decreased to 0.111, while the CI, which indicates the model’s ability to rank predictions, was 0.702. Although the CI value is lower than that of the model trained on the Kiba dataset, the performance remains commendable given the significantly smaller dataset size. Furthermore, the R² value increased to 0.833 and the PCC reached an impressive 0.930, highlighting a strong positive linear relationship between predicted and observed interaction values in the AMPK dataset (Table 2). The model outperformed commonly used machine learning models across all evaluation metrics (Supplementary Table S1). The strong performance across both datasets suggests that our deep learning architecture was effective. Additionally, the satisfactory performance on the AMPK dataset, despite its smaller size, indicates the effectiveness of transfer learning in improving model generalization.

**Fig. 2:**
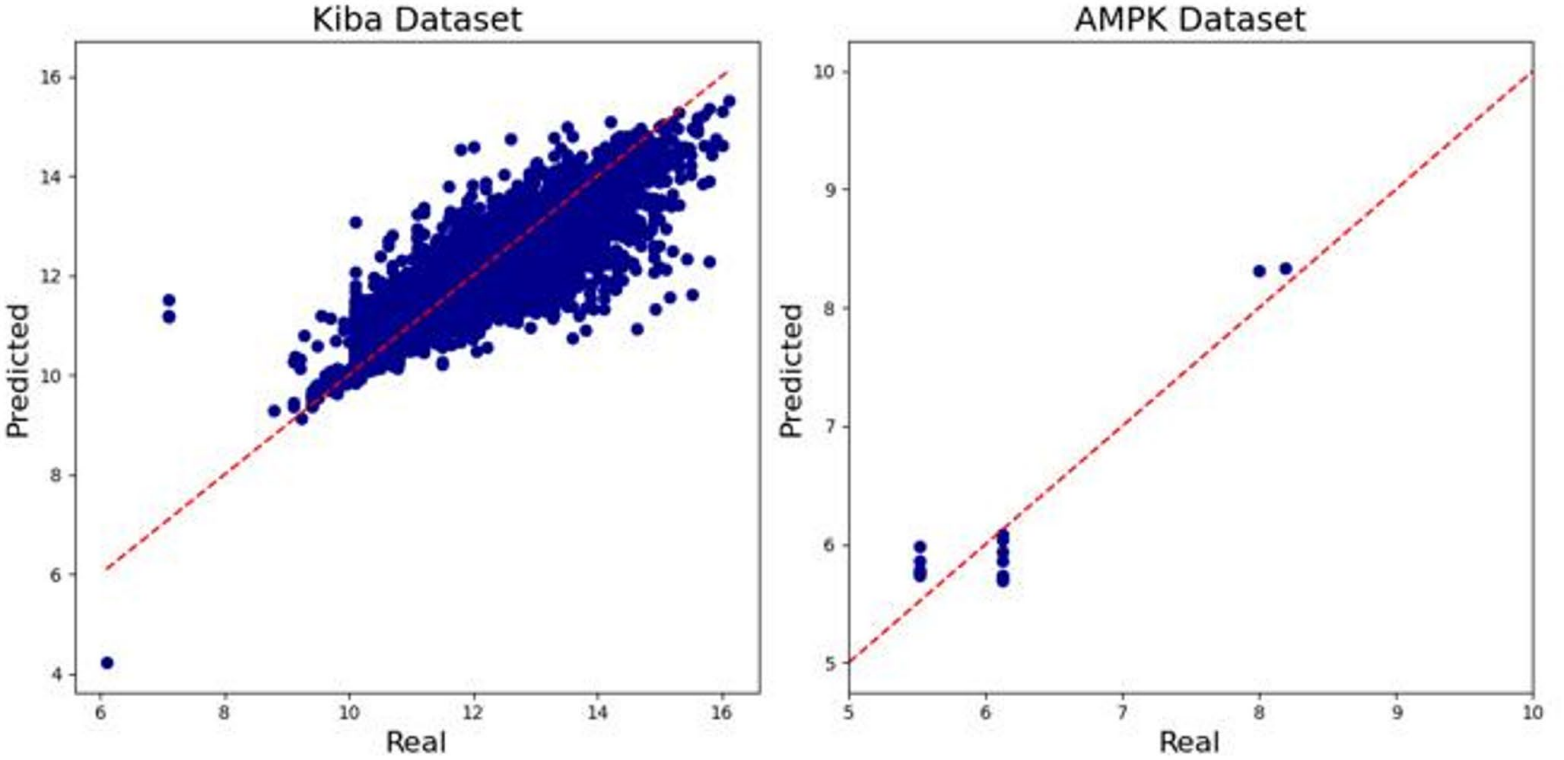
Predicted CPI values from the DL model against real CPI values for Kiba and AMPK test datasets (10% of entire dataset)

In Fig. 2, the predicted CPI values were compared against the actual CPI values for the test data within both the Kiba dataset and the AMPK dataset. The proximity of the test data to the x = y line represents the similarity between the predicted and actual CPI values. For the Kiba dataset, the test data points exhibited a close distribution around the x = y line, signifying that our pretrained model effectively predicted CPI values. For the AMPK dataset, the limited number of test data points made it challenging to observe a clear trend. However, the clustering of predictions around the x = y line indicates that the model demonstrated a reasonable accuracy in predicting CPI values despite the dataset’s smaller sample size.

**Table 2:** Performance of the DL model on Kiba and AMPK dataset under 5-fold cross validation

Datasets^1^	MSE (std)	CI (std)	*R*^2^ (std)	PCC (std)
Kiba	0.209 (0.004)	0.844 (0.001)	0.693 (0.006)	0.837 (0.001)
AMPK	0.111 (0.017)	0.702 (0.064)	0.833 (0.025)	0.930 (0.010)

^1^To show the general performance of the model, all performance metrics are presented as averages of 5-fold cross validation with standard deviations in parentheses.

### Prediction of AMPK activating blue food compounds using deep learning and in silico approaches

To identify blue food compounds with potential AMPK-activating properties, we employed a combination of a transfer learning-based deep learning model and an in silico molecular docking approach. Initially, molecular fingerprints and descriptors were calculated for a total of 31,080 compounds, excluding those for which calculations were infeasible. Using the transfer learning model, designed to predict AMPK activators, CPI values between AMPK and blue food compounds were predicted. The compounds were then ranked based on their predicted pEC_50_ values, and the top 10% (3,108 compounds) were selected for further investigation.

These 3108 blue food compounds were subsequently subjected to in silico docking studies with the AMPK α2 subunit to evaluate their binding interactions. By applying stringent selection criteria—Qikprop #stars property below 5 and a docking score under − 7—a refined subset of 94 blue food compounds was identified. The #star property indicates the number of molecular properties falling outside the 95% range of known drugs [41]. The combined approach of deep learning-based of transfer learning and in silico docking not only prioritized of potential AMPK activators from blue food compounds but also streamlined the identification process for compounds with desirable in vitro properties. To assess their biological viability, in vitro validation experiments were conducted on five commercially available compounds selected from this subset (Sect. "Anti-adipogenic effect of the predicted blue food compounds")

**Fig. 3:**
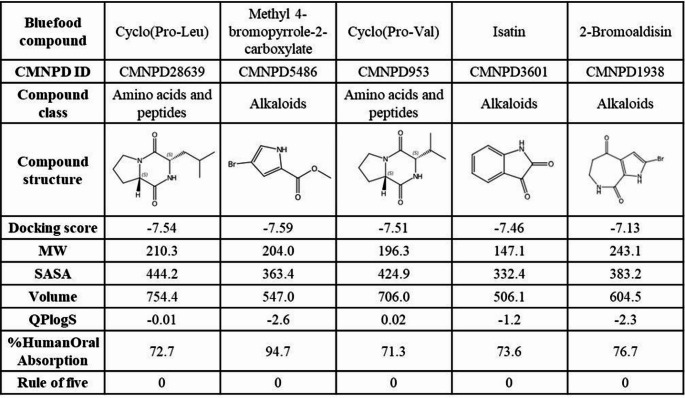
The calculated in silico molecular property of blue food compounds. The molecular property includes docking scores to AMPK and ADME properties (SASA, volume, QPlogS, %HumanOralAbsorption, Lipinski’s Rule of five)

### Absorption, distribution, metabolism, and excretion (ADME) property analysis and AMPK activity assay of the predicted blue food compounds

A high docking score alone is insufficient to determine the potential of predicted compounds as bioactive natural compounds. It is also essential to predict how these molecules interact within human metabolism. Therefore, we assessed the distribution of chemical and ADME properties of the predicted compounds (Fig. 3). The first set of parameters examined the chemical properties of the compounds, including molecular weight (MW), solvent-accessible surface area (SASA), and total solvent-accessible volume in cubic angstroms (volume). All compounds fall within acceptable ranges for SASA (300 to 1000) and MW (less than 500 Da). Next, predicted aqueous solubility (QPlogS) and human oral absorption (%) were evaluated as ADME properties. All predicted compounds exhibited QPlogS values within the acceptable ranges of -6.5 to -0.5 and human oral absorption between 25% to 80% [42] except for Methyl 4-bromopyrrole-2-carboxylate. Additionally, none of the predicted compounds violated Lipinski’s Rule of Five [43], further supporting their potential for oral bioavailability (Fig. 3). According to the ADMET properties calculated using SwissADME (Supplementary Table S2), all compounds showed a TPSA of approximately 90, indicating favorable oral absorption. Four of the compounds exhibited ideal drug-like lipophilicity with iLogP values between 1 and 3, whereas isatin had an iLogP below 1, suggesting higher hydrophilicity. All compounds were predicted to have high gastrointestinal absorption and were non-permeant to the blood–brain barrier. None of the compounds were predicted to inhibit CYP2C19, CYP2C9, CYP2D6, or CYP3A4. However, methyl 4-bromopyrrole-2-carboxylate and 2-bromoaldisin were predicted to be CYP1A2 inhibitors, indicating a potential risk of CYP1A2-mediated drug–drug interactions.

**Fig. 4:**
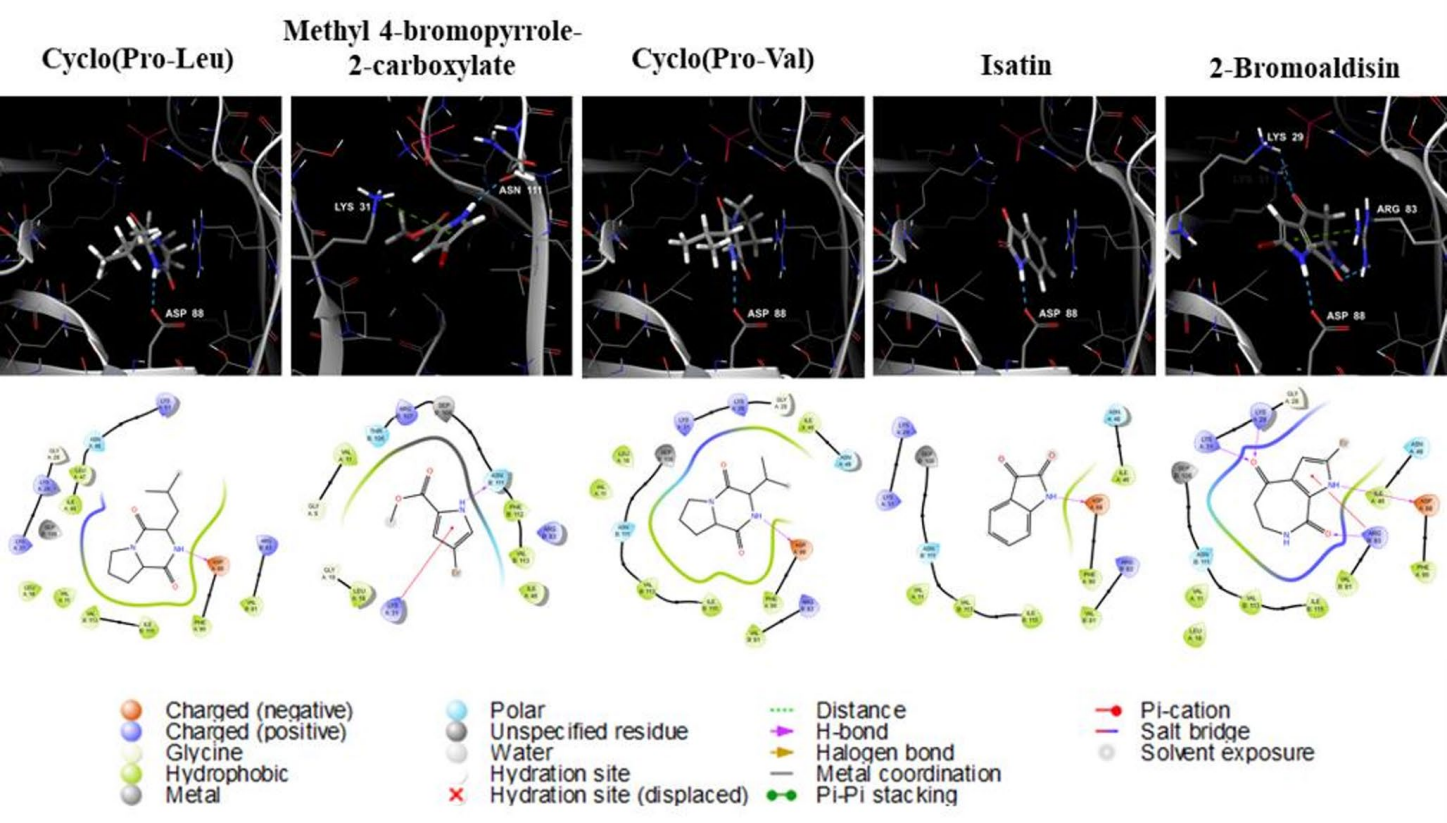
Docking diagrams (top) and 2D interaction diagrams (bottom) of the predicted blue food compounds with the AMPK α2 subunit

To gain further insights into binding interactions, 3D molecular representation and 2D ligand-protein interaction diagrams were analyzed. All five blue food compounds formed at least one hydrogen bond with the amino acids within the binding pocket of AMPK α2. Specifically, cyclo(Pro-Leu), cyclo(Pro-Val), and 2-bromoaldisin exhibited similar interactions, forming hydrogen bonds with Asp88 via their amino groups and engaging in broad hydrophobic interactions with residues such as Phe90, Ile115, and Val113. 2-Bromoaldisin also formed additional hydrogen bonds with Arg83, Lys31, and Lys29, along with a pi-cation interaction with Arg83. Isatin displayed a similar binding pattern, forming a hydrogen bond with Asp88 and engaging in hydrophobic interactions with several residues. Methyl 4-bromopyrrole-2-carboxylate established a hydrogen bond with Asn111 and a pi-cation interaction with Lys31 (Fig. 4).

Although all five blue food compounds exhibited comparable docking scores, AMPK activity assay revealed that three out of five compounds (cyclo(Pro-Leu), cyclo(Pro-Val), and isatin) have concentration-dependent AMPK activation (Supplementary Fig. S1). This discrepancy may stem from a limitation of docking-based predictions, which primarily evaluate static binding potential and do not fully account for the dynamic conformational and allosteric processes required for AMPK activation.

### Anti-adipogenic effect of the predicted blue food compounds

**Fig. 5:**
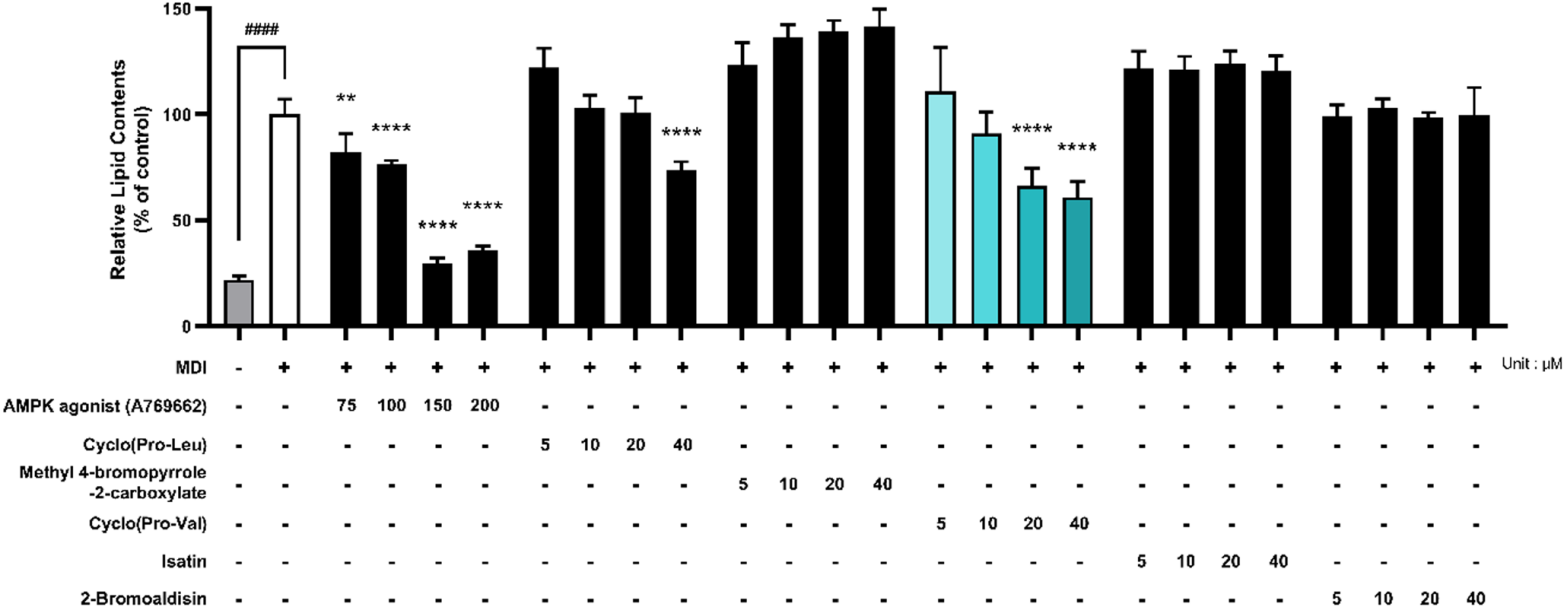
Evaluation of anti-obesity activity of blue food compounds. Effects of blue food compounds at different concentrations on the intracellular lipid accumulation in 3T3-L1 mature adipocytes. A769662, a known AMPK agonist, was included as a positive control at concentrations of 75, 100, 150, and 200 µM. The data are represented as mean ± standard error of the mean (*n* = 4). ####, significantly different from preadipocytes (Pre) by *p* < 0.0001. Significantly different from mature adipocyte (Ad) by * (*p* < 0.05), ** (*p* < 0.01), *** (*p* < 0.001), and **** (*p* < 0.0001)

To evaluate the anti-adipogenic effects of the predicted blue food compounds—cyclo(Pro-Leu), methyl 4-bromopyrrole-2-carboxylate, cyclo(Pro-Val), isatin, and 2-bromoaldisin—3T3-L1 preadipocytes were simultaneously treated with MDI and each compound at concentrations ranging from 5 to 40 µM. A769662, a well-characterized direct AMPK agonist, was included as a positive control at concentrations of 75, 100, 150, and 200 µM. MDI treatment significantly increased lipid accumulation compared to the undifferentiated control. A769662 significantly reduced lipid accumulation in a concentration-dependent manner, confirming the validity of the assay system. Among the five blue food compounds tested, cyclo(Pro-Val) significantly inhibited adipogenesis at concentrations of 20 µM and above, whereas cyclo(Pro-Leu) exhibited significant inhibition at 40 µM. The remaining three compounds—methyl 4-bromopyrrole-2-carboxylate, isatin, and 2-bromoaldisin—did not significantly inhibit adipogenesis at any of the tested concentrations. Among the five candidates, cyclo(Pro-Val) demonstrated the most potent anti-adipogenic effect, followed by cyclo(Pro-Leu) (Fig. 5).

### Anti-adipogenic effect of cyclo(Pro-Val) through AMPK activation

**Fig. 6:**
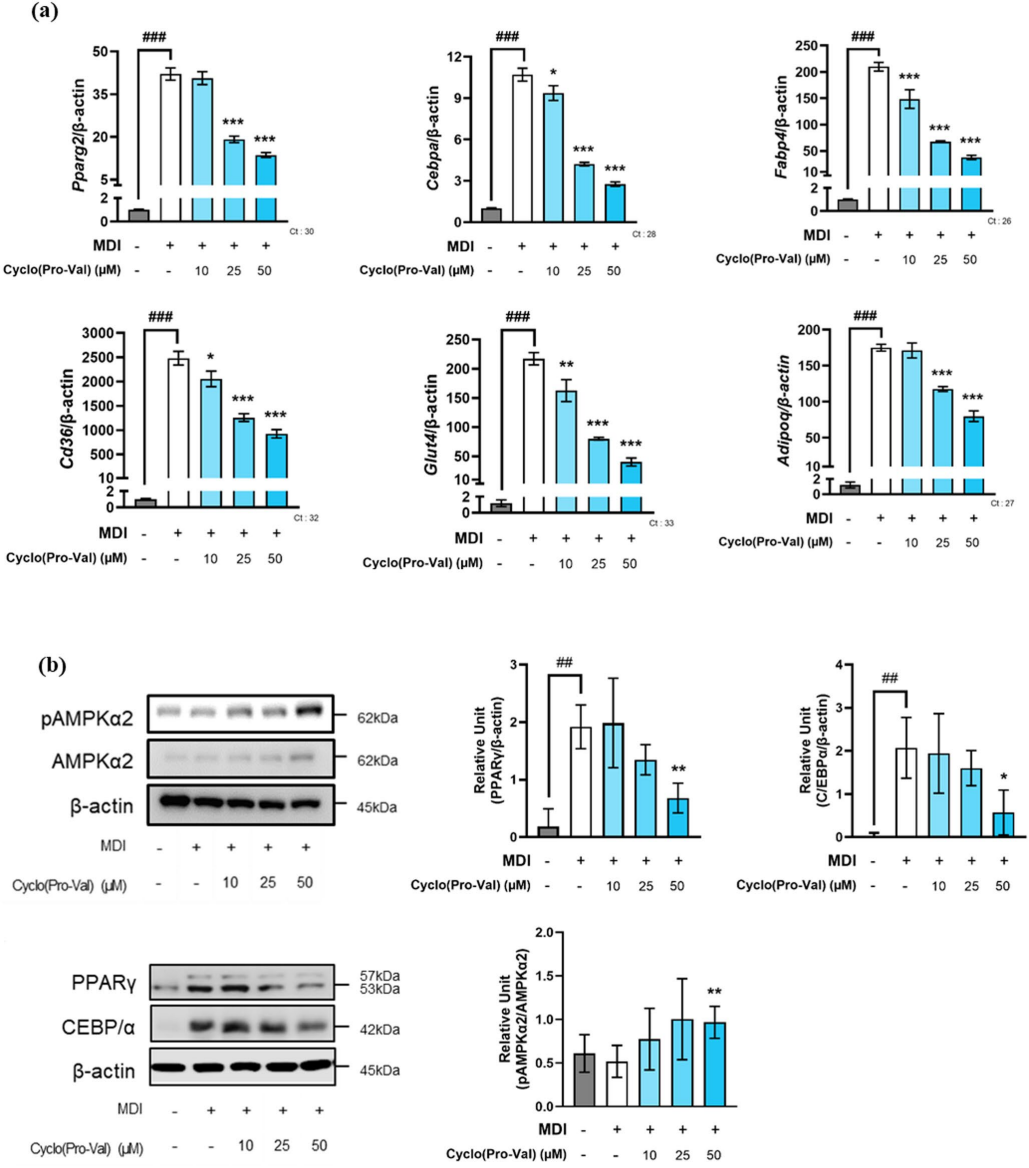
The anti-obesity effects of cyclo(Pro-Val) through AMPK activation. **a** The relative mRNA expression levels of the genes related to adipogenesis, lipogenesis, and gluconeogenesis in the 3T3-L1 mature adipocytes measured by qRT-PCR. The data are represented as mean ± standard error of the mean (*n* = 4). **b** The protein expression levels of PPARγ, C/EBPα, pAMPKα2 and AMPKα2 in 3T3-L1 mature adipocytes measured by Western blot analysis. The data are represented as mean ± standard error of the mean (*n* = 3 - 5). ### and ##, significantly different from preadipocytes (Pre) by *p* < 0.001 and *p* < 0.005, accordingly. Significantly different from mature adipocyte (Ad) by * (*p* < 0.05), ** (*p* < 0.01), and *** (*p* < 0.001)

To determine whether the identified anti-obesity compounds indeed inhibit adipogenesis via AMPK activation, we selected cyclo(Pro-Val) and examined its effects on adipogenic mRNA and protein expression levels. Cyclo(Pro-Val) was chosen due to its natural occurrence in the green algae *Ulva pertusa*, which is widely distributed along the coastal regions of Asia, making it readily accessible for further studies [44].To elucidate the mechanism by which cyclo(Pro-Val) inhibits lipid accumulation within adipocytes, we conducted quantitative PCR analyses focusing on genes involved in adipocyte differentiation, lipid metabolism, and glucose transportation (Fig. 6a). Cyclo(Pro-Val) treatment significantly reduced the expression of *PPARG2* and *CEBPA*, key transcription factors involved in adipogenesis, at concentrations of 25 µM and above. Correspondingly, the protein levels of PPARγ2 and C/EBPα consistently declined with increasing concentrations of cyclo(Pro-Val) (Fig. 6b). Additionally, cyclo(Pro-Val) suppressed the expression of adipogenesis-related genes, including *FABP4*, *CD36*, *GLUT4*, and *ADIPOQ* (Fig. 6a). Notably, cyclo(Pro-Val) increased the phosphorylation of AMPK α2, a crucial regulator of energy metabolism (Fig. 6b). These findings suggest that the anti-adipogenic effects of cyclo(Pro-Val) are mediated through the AMPK activation.

## Discussion

### Discovery of anti-obesity blue food compounds using combined deep learning and in silico approaches

Following the workflow depicted in Fig. 1, we identified 94 AMPK activators from a total of 31,080 blue food compounds through a systematic screening process. Utilizing a deep learning model in combination with in silico docking techniques significantly narrowed down the pool of candidates for subsequent in vitro validation. Among the five candidates subjected to in vitro validation, isatin had previously been recognized for its AMPK-activating properties [45]. For blue food compounds with no prior knowledge, their anti-obesity activity was confirmed for the first time. In silico docking analyses revealed that all five blue food compounds bind to the AMPK α2 active sites with varying binding affinities. These findings underscore the efficacy of our combined approach, which integrates deep learning models and in silico docking, in identifying bioactive blue food compounds. Similar workflow could be adapted to discover blue food compounds capable of modulating other target proteins beyond AMPK, potentially leading to novel treatments for various diseases.

Another key feature of the deep learning method developed in this study is the incorporation of transfer learning. The performance of deep learning is heavily influenced by the size of the training data. In general, larger datasets within a finite hypothesis class yield more accurate models. However, drug discovery studies often generate limited datasets due to the time-consuming and costly nature of experimental data acquisition. Transfer learning emerged as a powerful solution for overcoming data scarcity by leveraging knowledge from related datasets. Transfer learning refer to a technique where a model trained on one task is adapted to enhance performance on a different but related task by transferring learned knowledge or features from the initial training process [46]. This approach has been increasingly utilized in drug discovery studies to address low-data tasks, leading to successful outcomes [47, 48]. In this study, the dataset used to identify the AMPK α2 subunit activator consisted of only 245 data points, which is notably small. However, when a pre-trained model from the Kiba dataset was fine-tuned using transfer learning on the AMPK dataset, strong performance was observed across all evaluation metrics. Furthermore, this model was successfully applied to discover AMPK-activating blue food compounds, demonstrating its effectiveness. Given its success in predicting CPI values, this model is expected to be adaptable for predicting CPI in proteins with limited training data beyond AMPK.

Despite its effectiveness, our deep learning model has room for improvement. While it integrates both local and global features of compounds and proteins, additional features could further enhance compound and protein representation. For instance, incorporating biological pathways associated with proteins and toxicity information of compounds could potentially improve the model’s predictive performance. Additionally, the deep learning model used in this study suffers from the black box problem, as its prediction process is not fully interpretable. To address this issue, recent approaches such as attention mechanisms and SHAP (SHapley Additive exPlanations) [49] have been proposed, enabling a more transparent interpretation of deep learning model predictions.

Another advantage of our approach is the integration of in silico assessment to refine active compound candidates pre-selected by deep learning models, thereby further narrowing down the pool of candidates. In silico metrics used in this study included docking scores and the #stars property, which evaluate compounds’ protein interaction capabilities and druggability, respectively. The #stars property quantifies various ADME parameters that influence druggability, such as total solvent-accessible molecular surface area, aqueous solubility, and predicted binding constant to human serum albumin. It determines whether a compound’s properties fall within the 95% range of known drugs [41]. Through in silico modeling, we can not only assess the CPI strength but also evaluate the potential of the compound to act as an active agent upon ingestion. Consequently, by predicting both ADME properties and CPI strength in advance, this approach significantly streamlines the candidate selection process, which would otherwise require extensive in vivo studies.

### In silico molecular docking insights into AMPK and predicted blue food compound interactions

Notably, three compounds which showed concentration-dependent AMPK activation activity formed hydrogen bonds with Asp88, suggesting that this binding interaction is crucial for AMPK activation. Previous studies have shown that the well-known AMPK direct activator A-769,662 forms hydrogen bonds with Asp88 and Lys29, as well as pi-cation interactions with Arg83 and Ser108. Additionally, A-769,662 interacts with residues such as Val11, Gly19, Lys31, Ile46, Val81, and Val113 [50]. In our study, compounds like cyclo(Pro-Val) and cyclo(Pro-Leu), which demonstrated significant in vitro anti-obesity activity, exhibited similar interaction patterns. Cyclo(Pro-Val) formed a hydrogen bond with Asp88 and interacted with amino acids including Arg83, Ser108, Val11, Lys31, Ile46, Val81, and Val113. Similarly, cyclo(Pro-Leu) formed a hydrogen bond with Asp88 and interacted with Val11, Ile46, Val113 (Fig. 4). Despite its similar docking profile to A-769,662, 2-bromoaldisin showed no significant activity in both enzyme and anti-obesity assays. Its lack of efficacy likely stems from excessive hydrogen bonding and the steric hindrance of the bromine group, which may have rigidified the binding pocket and hindered the conformational changes necessary for AMPK α2 activation. These findings suggest that the blue food compounds identified in our study may bind to AMPK in a manner similar to A-769,662, interacting with the protein in comparable ways. A-769,662, has been shown to reduce weight gain and lower liver and plasma triglyceride levels by increasing fatty acid oxidation and decreasing fatty acid synthesis in vivo [51]. Additionally, it significantly hinders the differentiation of 3T3-L1 cells by down-regulating adipogenesis-related transcription factors and markers, such as PPARγ and C/EBP families.

### Activation of AMPK by cyclo(Pro-Val)

In this study, we employed a combined in silico and deep learning approach to identify 94 potential AMPK activating blue food compounds with a particular focus on cyclo(Pro-Val). Our findings indicate that cyclo(Pro-Val) inhibits adipogenesis by activating AMPK, a central regulator of cellular energy homeostasis. Activation of AMPK initiates a cascade of signaling events that modulate adipogenesis and various metabolic processes, including lipid and glucose metabolism [9]. This was evidenced by an increase in the ratio of phosphorylated AMPK to total AMPK upon treatment with 50 µM cyclo(Pro-Val). During the initiation of adipogenesis, the expressions of transcription factors such as PPARγ and C/EBPα increase, leading to the expression of genes associated with adipocyte maturation and lipid metabolism. However, activation of AMPK by compounds like cyclo(Pro-Val) inhibits the expressions of PPARγ and C/EBPα, thereby suppressing adipogenesis [13]. Additionally, cyclo(Pro-Val) downregulates the expression of adipocyte-specific markers, including *FABP4*, *CD36*, *GLUT4*, and *ADIPOQ* in a concentration-dependent manner. These findings underscore the critical role of AMPK activation as an upstream regulator of adipogenesis and suggest that targeting AMPK could be a promising strategy for controlling obesity.

At the tissue level, AMPK plays a crucial role in regulating metabolism in white adipose tissue (WAT). Activation of AMPK inhibits lipogenesis by phosphorylating and inhibiting ACC, thereby reducing the rate of fatty acid synthesis [52]. Moreover, AMPK activation suppresses lipolysis, the breakdown of triglycerides, which is essential for providing fatty acids and glycerol during fasting. This inhibition of lipolysis occurs through various mechanisms, including the direct inhibition of β-adrenergic-induced lipolysis [53]. Notably, while AMPK activation stimulates glucose transport by promoting GLUT4 translocation in muscle cells [54] its effects in adipocytes vary depending on the type of activator and cell line [55]. For instance, activation of AMPK by AICAR has been shown to decrease insulin-stimulated glucose uptake in both rat and 3T3-L1 adipocytes. However, the impact on basal glucose uptake varied, with an increase observed in 3T3-L1 adipocytes and a decrease in rat adipocytes [56–58]. While both AICAR and A-769,662 inhibited basal and insulin-stimulated glucose uptake across all models tested, the more potent and specific activator, 991, did not affect basal glucose uptake despite AMPK activation [59]. Furthermore, studies by Wu et al. [60] have demonstrated that deficiency of AMPKα in adipocytes leads to impaired thermogenesis in response to cold exposure and obesity under nutrient overload. Conversely, sustained activation of AMPK with A-769,662 promotes the browning of WAT in the inguinal region and offers protection against high-fat diet-induced obesity and associated metabolic disorders [60]. Overall, AMPK activation in adipocytes and WAT helps maintain energy homeostasis by regulating lipid and glucose metabolism, highlighting its importance in metabolic control.

## Conclusion

In this study, we introduced an innovative approach that integrates deep learning *and in silico* methods to efficiently identify anti-obesity compounds from the blue foods natural product repository. By leveraging a deep learning model trained on CPI, combined with in silico molecular docking, we successfully pinpointed 94 promising candidates targeting the AMPK a2 subunit. Subsequent in vitro validation of five commercially available compounds demonstrated their efficacy in inhibiting adipogenesis, underscoring the potential of our approach in accelerating the discovery of novel preventive therapeutic strategies for obesity and related metabolic disorders. Notably, the deep learning framework e developed is adaptable and can be extended to other diseases, broadening its applicability. Moreover, the AMPK activators identified in this study may have potential applications beyond obesity, suggesting possibilities for their utility in preventing and treating various diseases. In summary, our integrated deep learning and in silico approach not only streamlines the identification of bioactive compounds but also opens new avenues for preventive and therapeutic interventions across a spectrum of diseases.

## Supplementary Information

Below is the link to the electronic supplementary material.

Supplementary Material 1

## Data Availability

No datasets were generated or analysed during the current study.
