# Supplementary material for: Discovering anti-obesity blue food compounds via combined deep learning and in silico approaches
Source: Mol Divers. 2026 Mar 18;30(2):2889–904. doi: 10.1007/s11030-026-11506-5 (PMC13139301; doi:10.1007/s11030-026-11506-5)
Supplement: Supplementary file 1 — Supplementary Material 1 [file 11030_2026_11506_MOESM1_ESM.pdf]

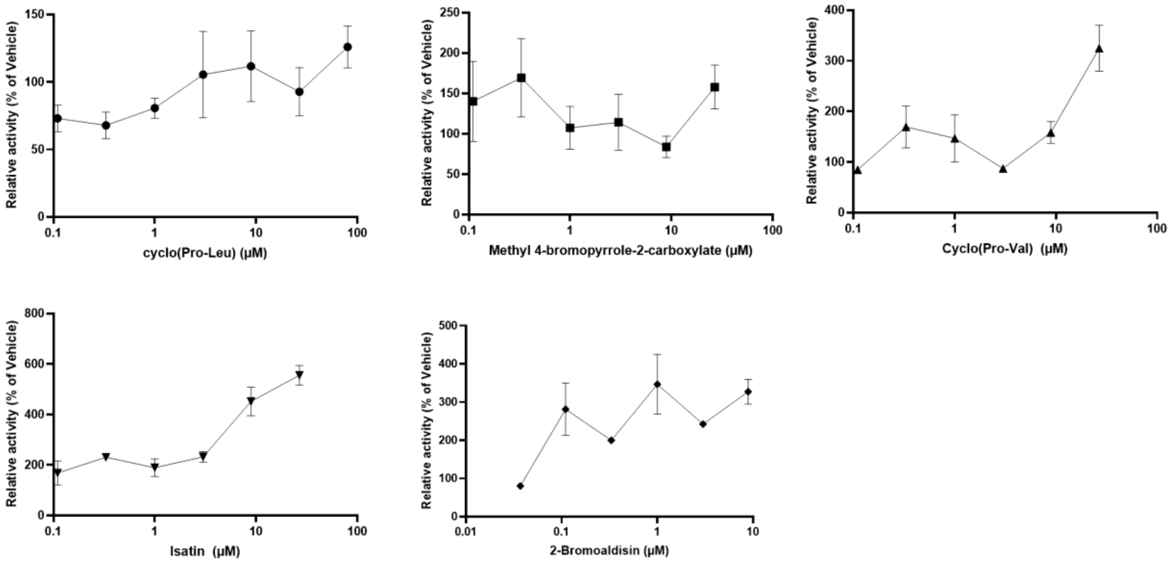

Figure S1. Activation effect of blue food compounds on heterotrimeric AMPK α2β1γ1 enzyme. Kinase activity assay was performed in triplicates the data are represented as mean ± SEM.

Table S1. Performance of the DL model and baseline machine learning models (values shown as mean ± standard deviation).

Model	MSE (std)	CI (std)	R^2 (std)	PCC (std)
**DL model**	**0.111 (0.017)**	**0.702 (0.064)**	**0.833 (0.025)**	**0.930 (0.010)**
Lasso	0.387 (0.127)	0.603 (0.042)	0.303 (0.036)	0.844 (0.059)
Random Forest	0.172 (0.096)	0.825 (0.028)	0.694 (0.100)	0.849 (0.057)
Gradient Boosting	0.158 (0.040)	0.774 (0.050)	0.704 (0.059)	0.892 (0.033)
SVR	0.571 (0.186)	0.619 (0.021)	-0.023 (0.020)	0.139 (0.038)
KNN	0.303 (0.115)	0.737 (0.067)	0.454 (0.079)	0.836 (0.078)

Table S2. ADMET evaluation of blue food compounds using SwissADME

Molecule	TPSA	iLOGP	ESOL Class	GI absorption	BBB permeant	CYP1A2 inhibitor	CYP2C19 inhibitor	CYP2C9 inhibitor	CYP2D6 inhibitor	CYP3A4 inhibitor	Lipinski #violations	Synthetic Accessibility
Cyclo(Pro-Leu)	49.41	2.26	Very soluble	High	No	No	No	No	No	No	0	2.44
Methyl 4-bromopyrrole-2-carboxylate	61.96	1.31	Soluble	High	No	Yes	No	No	No	No	0	2.19
Cyclo(Pro-Val)	49.41	2.03	Very soluble	High	No	No	No	No	No	No	0	2.34
Isatin	46.17	0.83	Very soluble	High	No	No	No	No	No	No	0	1.34
2-bromoaldisin	42.09	1.91	Soluble	High	Yes	Yes	No	No	No	No	0	1.81
